# Associations and Disease–Disease Interactions of COVID-19 with Congenital and Genetic Disorders: A Comprehensive Review

**DOI:** 10.3390/v14050910

**Published:** 2022-04-27

**Authors:** Altijana Hromić-Jahjefendić, Debmalya Barh, Cecília Horta Ramalho Pinto, Lucas Gabriel Rodrigues Gomes, Jéssica Lígia Picanço Machado, Oladapo Olawale Afolabi, Sandeep Tiwari, Alaa A. A. Aljabali, Murtaza M. Tambuwala, Ángel Serrano-Aroca, Elrashdy M. Redwan, Vladimir N. Uversky, Kenneth Lundstrom

**Affiliations:** 1 Department of Genetics and Bioengineering, Faculty of Engineering and Natural Sciences, International University of Sarajevo, Hrasnicka Cesta 15, 71000 Sarajevo, Bosnia and Herzegovina; 2 Institute of Integrative Omics and Applied Biotechnology (IIOAB), Nonakuri, Purba Medinipur 721172, India; 3 Department of Genetics, Ecology and Evolution, Institute of Biological Sciences, Federal University of Minas Gerais, Belo Horizonte 31270-901, Brazil; lucasgabriel388@gmail.com (L.G.R.G.); sandip_sbtbi@yahoo.com (S.T.); 4 Department of Biochemistry and Immunology, Institute of Biological Sciences, Federal University of Minas Gerais, Belo Horizonte 31270-901, Brazil; ceciliahrp@gmail.com; 5 Department of Bioinformatics, Institute of Biological Sciences, Federal University of Minas Gerais, Belo Horizonte 31270-901, Brazil; jessicaligia.pm@gmail.com; 6 Department of Physiology and Biophysics, Pharmacology, Institute of Biological Sciences, Federal University of Minas Gerais, Belo Horizonte 31270-901, Brazil; afolabi.oladapo9@gmail.com; 7 Department of Pharmaceutics and Pharmaceutical Technology, Faculty of Pharmacy, Yarmouk University, P.O. Box 566, Irbid 21163, Jordan; 8 School of Pharmacy and Pharmaceutical Science, Ulster University, Coleraine BT52 1SA, UK; m.tambuwala@ulster.ac.uk; 9 Biomaterials and Bioengineering Laboratory, Centro de Investigación Traslacional San Alberto Magno, Universidad Católica de Valencia San Vicente Mártir, c/Guillem de Castro 94, 46001 Valencia, Spain; angel.serrano@ucv.es; 10 Department of Biological Science, Faculty of Science, King Abdulaziz University, Jeddah 21589, Saudi Arabia; rredwan@gmail.com; 11 Therapeutic and Protective Proteins Laboratory, Protein Research Department, Genetic Engineering and Biotechnology Research Institute, City for Scientific Research and Technology Applications, New Borg EL-Arab 21934, Alexandria, Egypt; 12 Department of Molecular Medicine and USF Health Byrd Alzheimer’s Institute, Morsani College of Medicine, University of South Florida, Tampa, FL 33612, USA; vuversky@usf.edu; 13 PanTherapeutics, Route de Lavaux 49, CH1095 Lutry, Switzerland

**Keywords:** COVID-19, congenital anomalies, disease incidence and association, genetic diseases, genetic susceptibility

## Abstract

Since December 2019, the COVID-19 pandemic, which originated in Wuhan, China, has resulted in over six million deaths worldwide. Millions of people who survived this SARS-CoV-2 infection show a number of post-COVID complications. Although, the comorbid conditions and post-COVID complexities are to some extent well reviewed and known, the impact of COVID-19 on pre-existing congenital anomalies and genetic diseases are only documented in isolated case reports and case series, so far. In the present review, we analyzed the PubMed indexed literature published between December 2019 and January 2022 to understand this relationship from various points of view, such as susceptibility, severity and heritability. Based on our knowledge, this is the first comprehensive review on COVID-19 and its associations with various congenital anomalies and genetic diseases. According to reported studies, some congenital disorders present high-risk for developing severe COVID-19 since these disorders already include some comorbidities related to the structure and function of the respiratory and cardiovascular systems, leading to severe pneumonia. Other congenital disorders rather cause psychological burdens to patients and are not considered high-risk for the development of severe COVID-19 infection.

## 1. Introduction

Birth defects, also known as congenital diseases, can be inherited, or caused by environmental circumstances, leading to physical, intellectual, or developmental disabilities (https://www.who.int/news-room/fact-sheets/detail/congenital-anomalies accessed on 9 December 2021). Some birth defects can be fatal if they are not detected and treated early enough. Thousands of distinct birth abnormalities have been uncovered. According to the Centers for Disease Control and Prevention (CDC), birth defects are the most significant cause of death for newborns in the United States. In general, there are two categories of birth defects: structural and functional. Structural disorders relate to structural abnormalities of particular body parts. Cleft lip and palate, heart defects such as missing or misshaped valves, deformed limbs such as clubfoot, neural tube defects such as spina bifida, and issues related to the brain and spinal cord growth and development are just a few examples. Problems with the way body parts or systems work are referred to as functional or developmental dysfunctions. These problems often lead to intellectual and developmental disabilities (IDDs). They can include nervous system or brain dysfunctions, sensory problems, metabolic disorders, and degenerative disorders. Muscular dystrophy and X-linked adrenoleukodystrophy, which affect the nervous system and adrenal glands, are examples of degenerative illnesses. Both structural and functional problems are present in some birth malformations (https://www.nichd.nih.gov/health/topics/birthdefects/conditioninfo/causes accessed on 9 December 2021). Genetic or chromosomal problems, exposure to certain medications or chemicals, or certain infections during pregnancy can all cause birth malformations (https://ug1lib.org/book/5586785/4989ce accessed on 9 December 2021). According to the CDC, one out of every 33 newborns in the United States has birth defects. Birth abnormalities can occur at any time during pregnancy; however, some pregnancies are at a higher risk than others due to a shortage of folic acid, alcohol and tobacco use, drug addiction, and numerous diseases (https://www.nichd.nih.gov/health/topics/birthdefects/conditioninfo/types accessed on 9 December 2021).

Severe acute respiratory syndrome-coronavirus-2 (SARS-CoV-2) is a highly transmissible and deadly coronavirus that first appeared in late 2019. It triggered a pandemic of an acute respiratory disease known as coronavirus disease 2019 (COVID-19), which poses a hazard to global public health and safety [1]. The SARS-CoV-2 pandemic has been in focus since 2019. Studies have reported an association between viral infection and pre-existing congenital disorders. On the other hand, most instances, however, concern the implications of SARS-CoV-2 infection in patients who already have a congenital disorder [2].

Pre-existing (comorbid) medical conditions are associated with the severity of COVID-19, and more than 50 long-term effects of COVID-19 have been described [3]. It has also been reported that the COVID-19 related risk is increased with age, several comorbid conditions, cyanotic heart disease, and Body Mass Index (BMI) [4]. However, the association of COVID-19 with various congenital conditions has either been neglected or not frequently studied. This review tries to map the association between congenital and genetic disorders and their association with COVID-19.

## 2. Methods

**Figure 1:**
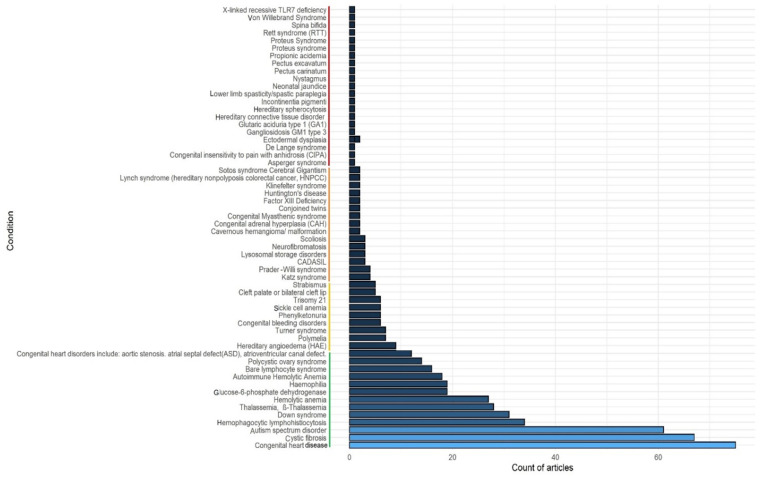
Congenital anomalies and genetic diseases associated with COVID-19 based on the PubMed literature search. Conditions having more than five PubMed hits are mostly considered in this review.

For this mapping, we mainly used two approaches. In the first approach, we searched the PubMed (www.pubmed.ncbi.nlm.nih.gov accessed on 7 March 2022) literature database from December 2019 to January 2022 using an advance search option (keywords in title) with the following keywords: (congenital [Title]) AND (COVID-19 [Title]), (Thalassemia [Title]) AND (COVID-19 [Title]), (Down Syndrome [Title]) AND (COVID-19 [Title]) etc., like more than 2980 congenital anomalies/genetic disorders from birth defect surveillance Toolkit of CDC (https://www.cdc.gov/ncbddd/birthdefects/surveillancemanual/resource-library/manual.html accessed on 7 March 2022). The number of articles with the name or synonym of congenital anomalies or genetic diseases and COVID-19 in the article’s title was recorded. The conditions showing more than five articles were first considered, to understand which anomalies or diseases are more frequently associated with COVID-19 (Figure 1). Once this mapping was completed, abstract level selection and curation was carried out following the PRISMA method to identify the articles having direct association and disease–disease-interactions with COVID-19.

In the second approach, we tried to identify those congenital anomalies and genetic diseases associated with COVID-19 as predicted by Barh et al., 2021 [5], but were not reported in the supporting primary literature at that time. We considered the work of Barh et al., 2021, as it was the first bioinformatics-based prediction to show association and disease–disease interactions between congenital or genetic disorders and COVID-19 with high precision.

## 3. Literature Search Results

**Figure 2:**
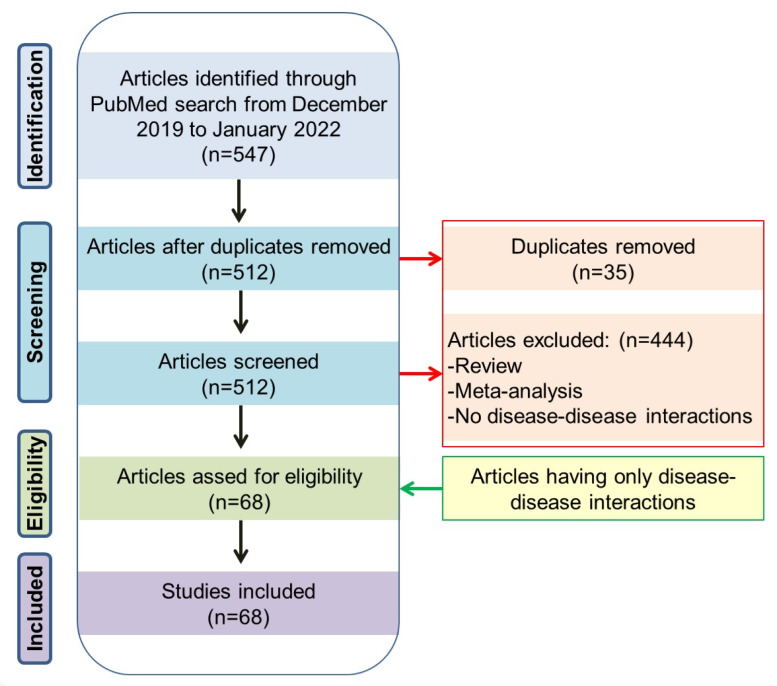
PRISMA flow diagram of the literature search and article selection to develop the key sections (direct association and disease–disease interactions between a congenital anomaly or genetic disease and COVID-19) of this review.

In the PubMed search, we found a total of 547 articles describing various congenital anomalies and genetic diseases and their associations with COVID-19. Based on analysis of these 547 publications, we found that congenital heart disease, cystic fibrosis, autism spectrum disorder, Downs’s syndrome etc., were the most reported cases associated with COVID-19 (for details, see Figure 1). Following the methods, finally a total 68 articles describing direct association and disease–disease interactions between the congenital anomaly or genetic disease and COVID-19 were considered to develop the main sections of the review (Figure 2). Additional references were used to describe various pieces of basic information on congenital anomalies or genetic diseases.

## 4. Most Frequently (>10 PubMed Hits) Associated Congenital and Genetic Disorders with COVID-19

### 4.1. COVID-19 and Congenital Heart Disease (CHD)

Congenital heart disease (CHD) showed a total of 75 PubMed hits in our literature search (Figure 1). Heart disease was identified as a risk factor for mortality in the early stages of the COVID-19 pandemic [6]. Adults with CHD, which affects more than 1.5 million people in the United States [7] and 2.5 million in Europe [8], are recognized as a high-morbidity population, susceptible to a variety of cardiovascular deterioration and dysfunction. As a result, these individuals are widely seen as high-risk, and they must adapt to a highly limited lifestyle and career choices. A recent clinical study involving 105 patients has indicated that patients with congenital cardiac defects such as cyanotic lesions, and unrepaired cyanotic defects (Eisenmenger syndrome), were at much higher risk if infected by SARS-CoV-2 [4]. The ACE2 receptor has been demonstrated to have a greater affinity for SARS-CoV-2 than for SARS-CoV. This, along with the presence of ACE2 on myocardial cells, may help explain how COVID-19 impacts the cardiovascular system (CVS) by accessing these cells and causing direct toxicity. 

Studies have indicated that COVID-19 patients with pre-existing cardiovascular disease (CVD) have a higher mortality rate than those with chronic obstructive pulmonary disease (COPD) [9]. The exact mechanism by which SARS-CoV-2 harms cardiomyocytes is unknown. It has been claimed that it could happen due to COVID-19-generated cytokine storm [10]. Another suggested explanation is that during acute respiratory distress syndrome (ARDS), the increased oxygen demand of cardiomyocytes occurs in a condition of hypoxia, resulting in oxidative stress [10]. Although there is no evidence to show how COVID-19 affects CHD patients, present research implies that they may be at an elevated risk of cardiovascular problems and intensive care unit (ICU) admission. The majority of cardiovascular problems occur in CHD patients with confirmed COVID-19, rather than suspected COVID-19 cases, with heart failure (55%), arrhythmias (22%), and stroke (22%) being the most common [11]. There has been no research reported on CHD repair surgery and the risks of perioperative COVID-19. However, a recent study of postoperative mortality in patients with perioperative SARS-CoV-2 infection found that overall mortality was 34% and that 94.1% of patients undergoing cardiac surgery had pulmonary problems. Male gender, age 70 or older, physical status classification system (ASA) grades 3–5, surgery for malignant disease, emergency surgery, and major surgery were all identified as risk factors [12]. Another study, focusing on children, recommends a surgical priority classification depending on the type of congenital cardiac abnormality. There are three types of operations: emergency (24–48 h), urgent (one–two weeks), and elective (more than two weeks) [13]. In conclusion, CHD patients of an advanced age with obesity and multiple comorbidities are at an increased risk when infected with SARS-CoV-2 [4], although the pediatric population is mainly protected. However, as the hazards are unknown, consenting these patients for surgery must be carried out cautiously [9].

### 4.2. COVID-19 and Cystic Fibrosis (CF)

In our literature search, Cystic fibrosis (CF) was the second most common genetic disease associated with COVID-19 described in 67 PubMed articles. CF is the most frequent fatal genetic illness in North America and possesses a classic Mendelian autosomal recessive trait [14]. The inheritance of two defective copies of the *CF transmembrane conductance regulator (CFTR)* gene has been linked to CF. Abnormal salt and water transport across epithelial surfaces is associated with abnormal *CFTR*. It presents itself in the lungs as mucus build-up and an inability to remove or clear inhaled organisms, leading to persistent infection and inflammation, which causes airway remodeling and illness. *CFTR* is also important in bicarbonate transport; abnormalities in its function cause the pH of the airway surface fluids to drop [14]. The recent COVID-19 pandemic has had an impact on patients with comorbidities. However, it is unclear if individuals with CF are more susceptible to COVID-19 or its negative repercussions. The role of recurring respiratory virus infections in disease perturbation and pulmonary exacerbations has made this a hot issue [15]. A search of the literature published between 28 April and 10 December 2020, was conducted using EMBASE and MEDLINE [16]. The goal of this search was to find publications that documented COVID-19 outcomes in people with a history of CF, who were infected by the virus. The incidence of SARS-CoV-2 infection was 0.07 percent in a global cohort study of 40 people with CF from eight countries, which is almost half of the 0.15 percent prevalence reported in the general population [17]. This evaluation included six studies that reported on a total of 339 people with CF who developed COVID-19. The European Cystic Fibrosis Society Patient Registry (ECFSPR) also contributed data on SARS-CoV-2 infections in 1236 individuals with CF from 30 countries (https://datawrapper.dwcdn.net/uQ1iZ/1/ accessed on 4 January 2022). Partial information was available from 946 (77%) of the cases. The most common age group was 18–29 years, with 56% having a FEV_1_ > 70 (Forced Expiratory Volume). Increased cough, fever, and exhaustion were the most prevalent symptoms. There were 582 persons with reported severities, with 550 (95%) being moderate or asymptomatic, 23 (4%) severe, and 9 (2%) catastrophic cases. In terms of treatment, 217 people (23%) were admitted to hospital, of which 30 (14%) were placed in the ICU. At the time of reporting, 866 (92%) of those infected had totally recovered, 39 (4%) were still infected, and 13 (1%) had died [16]. Although persons with CF are at risk for acute exacerbations of chronic lung disease, which are frequently caused by respiratory tract viral infections, available research suggests that SARS-CoV-2 infection rates in CF are lower than in the general population [16]. Nevertheless, there is evidence that specific subsets of the CF population, such as those who have had a transplant, may have a particularly severe clinical course. The low incidence of SARS-CoV-2 infection in the CF community is likely due to a combination of variables, including appropriate physical and social separation and the efficient application of established infection control concepts and practices emphasized as part of standard CF therapy [16]. 

### 4.3. COVID-19 and Autism Spectrum Disorder (ASD)

Autism spectrum disorder (ASD) emerged as the third most common congenital anomaly with 61 articles suggesting an association between ASD and COVID-19 (Figure 1). ASD is a neurodevelopmental disease marked by communication and social interaction difficulties and confined and repetitive patterns of behavior and interests. In 1943 Leo Kanner initially identified autism as a disease in youngsters with difficulties connecting to others and a high sensitivity to changes in their environment [18]. The Fifth Edition of the Diagnostic and Statistical Manual of Mental Disorders [19] introduced the broad diagnosis of ASD in 2013, combining four formerly distinct disorders: Autistic Disorder, Asperger’s Syndrome, Childhood Disintegrative Disorder, and Pervasive Developmental Disorder, which are some of the most common developmental disorders [19]. Families have had to alter their lifestyles as a result of the COVID-19 epidemic, including social isolation and parents working from home. The long-term effects of prolonged confinement on one’s mental health are unknown [20]. Changing routine can be difficult for children with ASD [21], and as a result, families with ASD children are more likely to experience anxiety and mental abnormalities during quarantine. A cross-sectional, observational, and analytical investigation was conducted. 

A total of 99 parents of school-aged children volunteered to participate in a study in April 2020 [22]. Parents of children with ASD and parents of children without neurodevelopmental conditions (control group; online form) were contacted by phone or email. An anonymous questionnaire with a total of 24 sets of questions was used to investigate the children’s demographic and clinical features, and the influence of social isolation at home during COVID-19 quarantine on various areas of the children’s and parents’ daily lives [22]. Of the 99 questionnaires collected, 43 were from children with ASD and 56 were from children who were not diagnosed with ASD. The age of children in the study was 10.75 ± 3.13 years old, with 68.7% of them being male. The majority of parents of children with ASD reported changes in their children’s behavior (72.1%), whereas most parents of children in the control group reported no changes (67.9%); the differences between the two groups were statistically significant (*p* = 0.05). Anxiety (41.7%), irritability (16.7%), preoccupation (11.1%), anger (5.6%), and impulsivity (2.8 %) were the most common causes of behavioral change indicated by parents of children with ASD. Parents of both groups felt that quarantine had more of a negative influence on learning than a favorable one (46.5% vs. 14% in the group of children with ASD and 50% vs. 19.6% in the control group) with no statistically significant differences between the two groups (*p* = 0.572). In addition, no statistically significant variations in the influence on cognitive development (*p* = 0.518) or familial ties (*p* = 0.298) were found [22]. The difference in the impact of quarantine on emotion management between the two groups was statistically significant (*p* = 0.02), and most parents of children with ASD reported a negative impact on emotion management (55.8%), in contrast to the parents of the control group. The latter mostly reported a positive or null impact on emotion management (71.4%). The greatest obstacles for children with ASD during this time were social isolation (41.4%), not being able to play outside (13.1%), changes in routine (11.1%), boredom (9.1%), and online education (7.1%), according to their parents. 

To summarize, there is strong evidence that children with ASD are more likely to experience anxiety, which can aggravate ASD symptoms and lead to more serious behavioral difficulties. Children with ASD experienced the most behavioral alterations throughout the school closure time in our study, while most children in the control group maintained their pre-quarantine conduct. Furthermore, anxiety, anger, preoccupation, hostility, and impulsive traits traditionally linked with this condition were indicated by parents as the causes of this behavioral change [22].

### 4.4. COVID-19 and Autoimmune Hemolytic Anemia (AIHA)

Hemolytic Anemia (AIHA) and COVID-19 gave 45 hits in our PubMed search (Figure 1). Anti-erythrocyte autoantibodies with or without complement activation induce an increase in the destruction of red blood cells (RBCs) in AIHA [23]. It can manifest itself in various ways, from asymptomatic to severe versions with catastrophic effects, and it can be idiopathic or due to the presence of another condition [23]. The first incidence of COVID-19 was reported in an AIHA patient in Sri Lanka [23]. A 23-year-old woman, who had been sick for three days with fever and cough, was treated in the Emergency Treatment Unit. Her COVID-19 quick antigen and PCR tests were both positive; thus, she was treated for a moderately severe SARS-CoV-2 infection in the ICU with non-invasive ventilation and high flow nasal oxygen (HFNO). Her COVID-19 exit PCR came back negative on day 20, and she was moved to the general medical unit to continue her treatment. She suffered significant weariness and shortness of breath while recovering from a moderate to severe COVID-19. Closer examination showed that she displayed a significant pallor, and tachycardia, and she was oxygen-dependent where her arterial blood gas showed no symptoms of hypoxia. Her ECG revealed a normal sinus rhythm, and she tested negative for troponin I. Initial blood tests indicated an abrupt reduction in hemoglobin from her normal level of 11 g/dL to 5.1 g/dL. The white blood cell and platelet levels were within normal ranges. There was no additional indication of heart or respiratory problems. The D-dimers were within an acceptable range, and the transthoracic echocardiography revealed normal left ventricular function with no signs of pulmonary embolism [23]. Her thyroid status was also normal. There was no sign of internal or external bleedings after a thorough examination. There was no history of hematological malignancy or connective tissue problems in the family.

In summary, before she acquired COVID-19, she was generally healthy with no risk indicators for AIHA. Her first blood tests revealed hemolysis, as evidenced by a high reticulocyte count, lactate dehydrogenase (LDH), and indirect hyperbilirubinemia. A blood smear established the existence of severe hemolytic anemia. The direct antiglobulin test was positive [23]. She was successfully treated with red blood cell transfusions and corticosteroid medication. After five days of therapy, the hemoglobin level had increased to 10 g/dL. Over three months, she was gradually weaned off prednisolone, and her hemoglobin was steady during the follow-up.

COVID-19-induced hemolysis has already been described in two articles. The first publication described a lady who needed steroids to treat her warm AIHA as she had underlying congenital thrombocytopenia [24]. The second study [25] included seven individuals who had symptomatic COVID-19 and exhibited warm or cold hemolysis symptoms nine days after arrival at the hospital. All of them required steroid therapy or a blood transfusion. In conclusion, AIHA coexistence with COVID-19 is a recently reported phenomenon that should be recognized in patients with unexplained or chronic anemia who have a current or recent history of COVID-19. Steroids showed a good response in the treatment of the first case of COVID-19 in the AIHA patient, which could be continued even after the COVID-19 acute phase had ended [23].

### 4.5. COVID-19 and Hematophagocytic Lymphohistocytosis (HLH)

Hematophagocytic lymphohistocytosis (HLH) is the fourth most frequent condition associated with COVID-19 (Figure 1). Hyperinflammation, cytokine storm, and secondary HLH (sHLH) are potential aggravating factors [26] for SARS-CoV-2 infections. Both non-COVID-19 and severely sick COVID-19 patients have shown significant sHLH death rates (about 40%) and viral infections are known to trigger sHLH (approximately 65%) [26]. Immunosuppression has been proposed as a therapeutic option, and preliminary findings are encouraging [27]. More data on the clinical treatment of sHLH in COVID-19 patients is critically needed in this scenario. Between April and May 2020, three patients were brought to an ICU in Vienna, Austria, according to the case report [26]. Real-Time qPCR was used for the detection of SARS-CoV-2 in pharyngeal and tracheal respiratory specimens. Repeated testing revealed positive results (Ct value > 35). The HScore was used to diagnose sHLH: Core temperature, hepato- and/or splenomegaly, number of cytopenias, triglycerides, fibrinogen, ferritin, and ASAT levels, history of immunosuppression, and (if possible) the presence of bone marrow hemophagocytes were all evaluated. For HLH, a positive result showed a sensitivity of 93% and specificity of 86%, and immunosuppressive therapy for sHLH was performed in a stepwise manner [28]. All three COVID-19 patients had cytokine storm, and their clinical course matched the clinical (fever, hypoxia, delirium) and laboratory (hyperferritinemia, lymphopenia, elevated IL-6, and CRP) characteristics of critically ill COVID-19 patients in general, as well as those with severe cardiac injury or coagulopathy [26]. The major cause for multiorgan failure is a rush of inflammatory cytokines, which leads to hyperinflammation and tissue destruction [27]. This shows that a significant number of severely ill COVID-19 patients have sHLH, which, if untreated, might explain the high fatality rates. Diagnosis of sHLH is difficult due to frequent misdiagnosis [26]. There are currently no randomized-controlled studies for sHLH therapy regimens, and evidence is poor even among non-COVID-19 patients. Immunomodulation has been proven to be effective in lowering death rates, and it was also recommended for COVID-19 treatment [27]. Immunosuppressive drugs like tocilizumab (IL-6 receptor monoclonal antibody) and anakinra (IL-1 receptor antagonist) may help against a cytokine storm. Furthermore, the data demonstrate that combining corticosteroids and immunoglobulins can help patients with severe COVID-19 [26,27,29]. In a case study for three patients, all patients had all of the symptoms of sHLH and were given a three-step treatment plan for sHLH induced by COVID-19 [28]. However, the identification of sHLH occurred too late for patients 1 and 2, but there was a favorable outcome for patient3—comparable to previously described results [26]. Routine screening for sHLH in COVID-19 patients using the HScore appears appropriate; patients who have a poor reaction to shock may be particularly vulnerable. A sequential therapy strategy using corticosteroids, immunoglobulins, and anakinra, as well as immunoadsorption, may help to mitigate the consequences of cytokine storms and may lower mortality [28].

### 4.6. COVID-19 in Adult Patients and Children with Down Syndrome (DS)

Down Syndrome (DS) or Trisomy 21 (T21) gave 37 PubMed hits and was the fifth most frequent congenital anomaly associated with COVID-19 (Figure 1). DS is the most prevalent chromosomal defect in humans. T21 causes immune dysregulation, which predisposes the individual to autoimmune disorders, as well as anatomical differences in the upper respiratory tract, such as a smaller trachea, enlarged adenoids/tonsils, glossoptosis, upper airway narrowing, and tracheal bronchus airway malacia, which all predispose the individual to a high frequency of respiratory diseases [30,31,32]. Despite the increased likelihood of respiratory infections, there has been no evidence of an increased risk of SARS-CoV-2 infection in DS patients. A case report of two COVID-19 cases in adult Caucasian patients with DS, who tested positive for SARS-CoV-2 without any respiratory coinfections after a nasopharyngeal swab (NFS), and a chest computer tomography (CT) scan revealed bilateral interstitial pneumonia [33]. A 59-year-old female, who had previously been diagnosed with congenital hydrocephalus, epilepsy, and hypothyroidism was the first case, while the second case involved a 42-year-old female who had hypothyroidism [33]. The clinical spectrum of DS is complex, with varied aspects affecting most organ systems. Various genetic processes may contribute to the phenotype of DS, and more than 80 clinical characteristics have been reported in DS, ranging in number and severity [34]. Case 1 had a more complex clinical DS with congenital hydrocephalus and epilepsy, while case 2 had a less complex clinical DS with hypothyroidism. The first patient was nearing the end of her DS lifespan, and an older adult is usually thought to present an additional independent risk factor for a poor outcome of SARS-CoV-2 infection. At the time of admission, both patients had lymphopenia. The first patient had a stable course of disease for ten days until provoked, requiring low-flow oxygen supplemental therapy but no substantial inflammatory index changes. The second patient’s inflammatory indexes and need for oxygen supplementation increased over time, requiring CPAP assistance and mechanical ventilation [33]. The laboratory parameters (leukocytes, lymphocytes, C-reactive protein, aspartate aminotransferase, alanine transaminase, total bilirubin, gamma-glutamyl transferase and D-dimer) were followed for both patients [33].

The cytokine release syndrome is frequently associated with severe respiratory impairment in COVID-19 patients, which is caused by an increased immunological response to the virus [35]. Those with severe COVID-19 experienced lymphopenia and high levels of circulating proinflammatory cytokines when compared to patients with moderate COVID-19 illness. Type I and III interferons (IFNs) appear to be involved in SARS-CoV-2 cytokine production, which is followed by immune cell infiltration and activation, cytokine hyperproduction, worsened immunological activation, and deterioration of progressive respiratory function [36]. In DS, studies have revealed an interferonopathic hallmark characterized by dysregulation of IFN-inducible cytokines, resulting in persistent inflammation and broad responsiveness to IFN activation across the immune system [37]. The first patient did not get immunomodulating medication since it was not yet available at the time of admission. However, the second patient was given sarilumab, the monoclonal antibody against IL-6, and her respiratory function gradually improved over the next few days until she recovered. Furthermore, it has been suggested that there may be mutations in other *type I IFN*–related genes in other patients with life-threatening COVID-19 pneumonia and the administration of type I IFN may be of therapeutic benefit in selected patients, at least early in the course of SARS-CoV-2 infection [38].

In COVID-19 patients, elevated levels of the inflammatory cytokine IL-6 were linked to a higher risk of death in retrospective studies, suggesting that it may operate as a crucial mediator for respiratory failure and shock [39]. Finally, due to the high number of comorbidities, structural changes in the upper respiratory tract, and immunological dysregulation, people with DS have a higher risk of respiratory infections and a poor prognosis. It is critical to recognize the therapeutic importance of DS in the treatment of COVID-19.

Additionally, two incidences of SARS-CoV-2 infection in children with DS have been reported [40]. The first case was a 14-year-old Caucasian girl with DS who had no cardiac abnormalities but was overweight (BMI 36) and had obstructive sleep apnea (OSA), which is prevalent in DS and can lead to pulmonary issues. In the second case, a 34-month-old North African child was diagnosed with DS and bilateral interstitial pneumonia, which needed 15 days of hospitalization and treatment with external oxygenation and antibiotics (ceftriaxone and azithromycin) [40]. She was readmitted after two weeks with high fever and skin rashes, which lasted three days, and antibiotic treatment with cefotaxime was started. She began to show signs of Kawasaki disease on the third day. Treatment began according to the Italian Society of Pediatrics’ standards, which included intravenous immunoglobulin, oral steroids, and high doses of aspirin, as well as a second dosage of immunoglobulin given due to a persistent fever. After this treatment, the fever went down, and aspirin administration was continued for another six weeks. SARS-CoV-2 serology (IgM, IgG) was positive. Follow-up visits revealed no problems in the heart or other organs [40]. 

Previous coronavirus epidemics have shown that these viruses have a lower proclivity for affecting youngsters. Only 6.9% of patients infected during the 2003 SARS-CoV outbreak were minors, and there were no fatalities among those under the age of 18. Furthermore, children were discovered to have a milder variant of the disease. Only 2% of the patients in the Middle East respiratory syndrome coronavirus (MERS-CoV) outbreak, which began in 2012 and is still ongoing, were youngsters [41,42]. Children appeared to be comparatively spared at the start of the COVID-19 pandemic, but later studies from multiple sites revealed a likely COVID-19-related severe multisystem inflammatory syndrome in children (MISC) and young adults [43,44]. The literature on COVID-19 in DS patients is still sparse, however, DS should be considered as a risk factor [45]. Children with DS have a distinct cardiovascular disease profile. Furthermore, anatomical abnormalities of the airways are substantial risk factors for respiratory infections in individuals with DS [40].

### 4.7. COVID-19 and Thalassemia

Thalassemia ranked the sixth most frequent genetic disease associated with COVID-19 (Figure 1). Patients with pre-existing chronic morbidities are more likely to be impacted by SARS-CoV-2 infection, but no data on Thalassemic Syndromes (TS) is available. TS and hemoglobin variations are one of the most common causes of anemia, affecting more than 7% of the global population, according to the WHO [46]. Thalassemia is divided into two types: transfusion-dependent thalassemia (TDT) and non-transfusion-dependent thalassemia (NTDT). Infections, primarily caused by bacteria, are a significant cause of death and morbidity in TS patients. Stress erythropoiesis, iron excess, splenectomy, and adrenal insufficiency are just a few of the factors that might enhance infection susceptibility [47]. In the study reported by Motta et al., an electronic Case Report Form (eCRF) survey in Italy was conducted to analyze the impact of SARS-CoV-2 infection on TS. Eleven cases of TS with COVID-19 had been collected as of 10 April 2020 [48]. The average age was 44.11 years (range 31–61 years), with females accounting for 55% (6/11). There were ten TDT patients and one NTDT patient. All patients had thalassemia-related comorbidities, eight had splenectomies, and one had sildenafil-treated pulmonary hypertension. In 55% (6/11) of patients, the likely source of infection was identified: two had contact with COVID-19 positive people, and four had occupational exposure (e.g., hospitals). Three of the patients had no symptoms. One patient was hospitalized with high fever, bone marrow hypoplasia, lymphopenia, and agranulocytosis (on deferiprone medication), where the third swab came back positive. Six of the eleven people were admitted to hospital, although none of them required mechanical breathing. The patient who required more intensive breathing support with continuous positive airway pressure (CPAP) had a history of diffuse large B-cell lymphoma (DLBLC), treated with chemotherapy a year earlier and is currently in complete remission. Only three of the six patients brought to hospital got purportedly COVID-19-specific treatment: one hydroxychloroquine (HCQ), one HCQ plus ritonavir/darunavir, and one HCQ plus anakinra [48]. Due to simultaneous amiodarone medication and a higher risk of life-threatening arrhythmia, one patient did not receive HCQ. The clinical program lasted from 10 to 29 days. Ten patients have recovered clinically and were receiving daily phone calls for follow-up. The presence of splenectomy in eight of eleven individuals did not appear to alter the clinical outcome. Except for the patient with myelosuppression, there was no increase in blood demand. When the NTDT patient’s luspatercept therapy was stopped, his hemoglobin dropped from 110 to 82 g/L, identical to the pre-luspatercept period. Death, severe SARS-CoV-2, or symptoms of cytokine storm were not found in these eleven participants, which is remarkable given their average age and the prevalence of significant comorbidities. Although early, these findings show that COVID-19 severity is not elevated in TS patients. More cases must be studied to determine the impact of SARS-CoV-2 and its outcome in these vulnerable people [48].

### 4.8. COVID-19 and G6P Dehydrogenase Insufficiency

The seventh most frequent condition associated with COVID-19 in our literature search was glucose-6-phosphate dehydrogenase (G6PD) insufficiency (Figure 1). The varied vulnerability to infection is one disconcerting characteristic of COVID-19. Some exposed individuals were asymptomatic, while others showed mild to moderate symptoms, and still others became critically ill and died. Hospitalization rates rose with age, and over 90% of hospitalized patients had underlying medical problems [49]. G6PD insufficiency is one issue to consider. Around 400 million individuals worldwide are affected by this X-linked recessive condition with multiple allelic variations, showing a higher frequency in Africa, the Mediterranean area, and Asia [49]. Decreased G6PD synthesis caused nicotinamide adenine dinucleotide phosphate deficiency and reduced glutathione, resulting in oxidative stress and red blood cell death [49]. Patients can develop hemolytic anemia due to some infectious agents and drugs, even if they are asymptomatic. There is evidence to imply an association between G6PD deficiency and greater susceptibility to SARS-CoV-2 infection, as well as the severity of the illness [50]. G6PD deficiency might increase the susceptibility to coronavirus infection. For example, G6PD-deficient cells infected by human coronavirus 229E showed considerably enhanced coronavirus gene expression and viral particle generation [51]. Furthermore, elderly people with G6PD deficiency are more likely to have red blood cells with lower G6PD levels, lower glutathione levels, and increased red blood cell turnover [52]. This may predispose older G6PD deficient patients to a lower threshold for hemolytic crises after exposure to a triggering event such as coronavirus infection. Chloroquine (CQ) has been observed to cause hemolysis in G6PD-deficient individuals, therefore it should be used with caution [53]. Its molecular variant, HCQ, an antimalarial medicine, has also been evaluated as a potential COVID-19 therapeutic. However, a recent study documented an acute hemolytic event in a COVID-19 patient with G6PD deficiency treated with HCQ [53]. In COVID-19 patients with G6PD deficiency, HCQ may enhance oxidative stress, potentially triggering hemolytic anemia [53]. More research is needed to clarify whether there is an association between G6PD deficiency and COVID-19 in increased susceptibility to infection and disease severity. This is significant for several reasons. First, it will enable the identification of a subgroup of COVID-19 patients, who will require intensive monitoring and supportive care. Second, some therapies, such as HCQ, may be contraindicated in these individuals. Third, the discovery of this link might lead to the development of new treatments for COVID-19, such as the use of antioxidants. Finally, those with known G6PD impairment will need this knowledge to guide their decisions and activities in order to avoid SARS-CoV-2 infections.

### 4.9. COVID-19 and Agammaglobulinemia

According to the CDC, patients with primary immunodeficiency diseases are prone to severe COVID-19. One case study [54] described a 28-year-old man diagnosed with X-linked Agammaglobulinemia (XLA), who had received a septic hip at the age of one year. B-cells were completely absent, and XLA was detected by flow cytometry. His medical history included a persistent multifocal cellulitis of his leg caused by a helicobacter species, which had been effectively treated without recurrence in 2019. His infectious history was otherwise normal, and he never received sequence confirmation of the *Bruton’s tyrosine kinase* gene, even though his diagnosis of XLA was highly suggestive based on his clinical history of early-onset infections and the lack of B-cells. He received 10 g of subcutaneous immunoglobulin (SCIG) on a weekly basis. He had a one-week history of fever, chills, hyposmia, and increasing productive cough and dyspnea when he arrived at the hospital. Prior to his arrival, he tested positive for COVID-19 by RT-PCR from a throat sample [54]. The patient was tachycardic, tachypneic, and required 2 L of oxygen through nasal prongs when admitted. A chest X-ray revealed bilateral airspace opacities, which might indicate pneumonia. Hyponatremia, leukopenia, thrombocytopenia, transaminitis, and increased inflammatory markers were also present in his blood. Despite these interventions, the patient’s hypoxia and breathing effort continued to deteriorate. He needed 5 L of oxygen via nasal prongs by day four and had a respiratory rate of 22. He was subsequently started on a five-day remdesivir regimen. On the fourth day, he self-administered his SCIG dosage. He was moved to the ICU on day five after requiring 60% oxygen via a high-flow nasal cannula. He was then given 500 mL of convalescent plasma. The patient began to show symptoms of clinical improvement on day nine, and his oxygen needs had decreased. He returned to the ward and was subjected to daily chest X-rays to monitor the development of a pneumothorax. He was weaned off oxygen by day 11, and the pneumomediastinum had cleared. On day 13, he was released [54]. Although it is hypothesized that patients with primary immunodeficiencies are at an increased risk of more severe infections due to COVID-19, few studies in the literature cover these situations [55]. Two individuals with Agammaglobulinemia (one with XLA) and five patients with common variable immunodeficiency (CVID), who tested positive for COVID-19 were documented in an early investigation [55]. Patients with Agammaglobulinemia had a much milder course than those with CVID. A second study of two XLA patients, who recovered quickly after being infected with SARS-CoV-2 and did not require critical care, backs this up [56]. According to international research, B-lymphocytes may be directly engaged in COVID-19-related inflammation, and their absence may result in lesser illness. When paired with the recent case series, the reported case [54] supports the idea that individuals without B-lymphocytes can nevertheless generate a significant inflammatory response to SARS-CoV-2 infection. In contrast to previous case reports, this study showed that XLA patients are still at risk of serious consequences from SARS-CoV-2 infection. Antibodies may be critical for viral neutralization, based on the quick recovery reported in XLA patients after receiving convalescent plasma. While the effect of remdesivir cannot be discounted, the patient’s quick response to convalescent plasma implies that humoral immunity is a key aspect in recovery from COVID-19. More research is needed to ascertain if the observed response to convalescent plasma is unique to individuals lacking B-lymphocytes [54].

### 4.10. COVID-19 and Polycystic Ovary Syndrome (PCOS)

Polycystic ovary syndrome (PCOS) is the 12th most common condition associated with COVID-19 (Figure 1). Women suffering from PCOS have lately been identified as an underserved and possibly high-risk group for COVID-19 [57]. PCOS is a lifelong metabolic disorder that is most commonly linked with androgen excess, infertility, and polycystic ovarian morphology based on ultrasound [58,59]. Insulin resistance is connected to low-grade chronic inflammation and androgen excess in PCOS, both of which influence adipocyte biology and metabolism. Owing to androgen excess in women with PCOS, the gender advantage due to estrogen’s preventive impact against COVID-19 appears to be lost [60]. In women with PCOS, insulin resistance with excess insulin or proinsulin promotes steroidogenesis. The Health Improvement Network (THIN) database was used to conduct a population-based retrospective closed cohort analysis to estimate the incidence risk of SARS-CoV-2 infection in women with PCOS compared to those without PCOS. THIN is a longitudinal primary care electronic medical records database including anonymized data from 365 current general practices in the United Kingdom [61]. The major question was whether the primary care physician categorized COVID-19 as suspected or confirmed. To calculate unadjusted and adjusted hazard risks (HR) of SARS-CoV-2 infection in women with PCOS compared to women without PCOS, the authors used a Cox proportional hazards regression model with stepwise inclusion of explanatory variables (age, BMI, impaired glucose regulation, androgen excess, anovulation, vitamin D deficiency, hypertension, and cardiovascular disease) [61]. The researchers found 21,292 women with a coded diagnosis of PCO/PCOS and chose 78,310 age- and practice-matched control women at random. In women with and without PCOS, the crude COVID-19 incidence was 18.1 and 11.9 per 1000 person-years, respectively. Women with PCOS have a 51% greater risk of COVID-19 than women without PCOS, according to age-adjusted Cox regression analysis (HR: 1.51 (95% CI: 1.27–1.80), *p* < 0.001). HR was lowered to 1.36 (1.14–1.63)] after correcting for age and BMI, *p* = 0.001. Women with PCOS had a 28% higher risk of COVID-19 (aHR: 1.28 (1.05–1.56), *p* = 0.015) in the fully adjusted model [61]. According to the study, women with PCOS have a higher risk of cardio-metabolic illness, which has been identified as a risk factor for COVID-19, and they should be urged to follow infection control measures during the COVID-19 pandemic [61].

## 5. Congenital and Genetic Conditions with Five to Ten PubMed Hits

### 5.1. COVID-19 and Hereditary Angioedema (HAE)

Hereditary angioedema (HAE) is the top condition associated with COVID-19 among conditions with less than ten PubMed ID hits (Figure 1). HAE, due to C1 inhibitor (C1INH) deficiency (HAE-C1INH), is a rare autosomal dominant and potentially life-threatening disease, clinically characterized by swelling attacks of the subcutaneous (SC) tissue and mucous membranes due to dysregulation of the plasma kallikrein-kinin system with enhanced generation of bradykinin [62]. In HAE-C1INH, the incidence and consequences of COVID-19 remain unclear. As ACE2, a key receptor protein involved in SARS-CoV-2 infectivity, also degrades kinins and bradykinin excess, it has been hypothesized to play a pathologic role in the severe respiratory complications of COVID-19 and individuals with HAE-C1INH have been hypothesized to be at increased risk for SARS-CoV-2 infection and complications [63]. The data for the reported study [64] came from online surveys, in which people and their families self-reported SARS-CoV-2 infection, complications and morbidity, were asked to fill out an online questionnaire. HAE patients with C1 inhibitor (C1INH) deficiency (HAE-C1INH), HAE patients with normal C1 inhibitor (HAE-nl-C1INH), and household controls were among the participants (controls). The effects of HAE medicines were investigated [64]. A total of 1162 individuals participated in the survey, including 695 persons with HAE-C1INH, 175 persons with HAE-nl-C1INH, and 292 healthy persons. The incidence of reported COVID-19 cases was not statistically different between normal controls (9%) and HAE-C1INH (11%) participants but was considerably higher in HAE-nl-C1INH (19%) subjects (*p* = 0.006). Obesity was shown to be positively associated with COVID-19 in the general population (*p* = 0.012), with a similar but non-significant trend in HAE-C1INH patients. Comorbid autoimmune illness was found to be a risk factor for COVID-19 in HAE-C1INH patients (*p* = 0.047). The severity and consequences of COVID-19 were similar across all groups. COVID-19 levels were lower in HAE-C1INH patients, who were treated with prophylactic subcutaneous C1INH (5.6%; *p* = 0.0371) or on-demand icatibant (7.8%; *p* = 0.0016). When compared to normal controls, persons with HAE-C1INH, who were not taking any HAE drugs had a higher incidence of COVID-19 (24.5%; *p* = 0.006) [64]. The findings revealed that participants with HAE-C1INH, who did not use HAE drugs, had a considerably higher risk of reported COVID-19 than normal controls, albeit no significant increase in COVID-19–related complications were found. SC pd–C1INH and icatibant usage were linked to a substantial decrease in COVID-19 levels in HAE-C1INH patients [64]. The participants with HAE-nl-C1INH reported more COVID-19 symptoms than the controls, but HAE medicines did not affect the likelihood of infection. All these findings suggest that the traditional complement and tissue kallikrein systems are involved in SARS-CoV-2 infection [64].

### 5.2. COVID-19 and Parsonage-Turner Syndrome (PTS)

Parsonage–Turner Syndrome (PTS) showed eight PubMed hits in our literature search (Figure 1). PTS, also known as idiopathic brachial plexopathy or neuralgic amyotrophy, is a rare illness with a documented overall incidence of 1.64 per 100,000 persons [65]. Young people are usually most affected, while incidences have been observed in children as young as three months old and in persons as old as 75 years of age. PTS is marked by sudden onset of significant shoulder discomfort, usually unilateral, that can spread to the arms and hands [65]. The clinical history/symptoms and physical examination results are used to diagnose PTS. Additional diagnostic testing, such as imaging and electromyography, may help confirm the diagnosis of PTS by ruling out alternative causes [65]. PTS has been documented in the postoperative, postinfectious, and post-vaccination settings, even though the origin and pathogenesis are unknown. As PTS variations are hereditary, there might be a genetic component. PTS has been linked to autoimmune diseases such as systemic lupus erythematosus, temporal arteritis, and polyarteritis nodosa [65,66]. Two possible explanations have been reported for PTS. Either a viral disease directly affects the brachial plexus or an autoimmune reaction to the viral infection or the viral antigen takes place [67]. Various viral, bacterial, and fungal diseases have been linked to PTS, but only two cases of SARS-CoV-2 infection have been confirmed.

The first case was a 17-year-old female patient who had been experiencing shortness of breath and joint discomfort for several weeks and had no substantial medical or surgical history. She claimed her symptoms started three months earlier, when she had fever, chills, and other upper respiratory infection symptoms for approximately a week [68], until they went away without treatment. A few weeks later, the patient complained of a new onset of multifocal joint discomfort, mostly affecting the left shoulder and left hand. The discomfort was described as persistent. However, it got worse by movement. Her symptoms improved dramatically after seeing her primary care physician and she was briefly treated with oral steroids. However, the patient began to feel increasing abdomen discomfort, shortness of breath, palpitations, and joint pain shortly after, prompting her to seek medical help [68]. A physical examination showed that her serum inflammatory indicators were significantly enhanced, and a SARS-CoV-2 RT-PCR test from a nasopharyngeal swab was negative, but serum IgG antibodies for SARS-CoV-2 were positive, indicating past infection/exposure. The supraspinatus, infraspinatus, teres minor, teres major, and trapezius muscles all showed uniformly elevated T2 signal on MR of the left shoulder, which was consistent with PTS diagnosis [68]. Although the patient’s history and physical examination are important for the diagnosis of PTS, imaging is typically used to rule out other etiologies for a patient’s symptoms and confirm a suspected diagnosis of PTS. The imaging method of choice for evaluating PTS is MRI [68].

The second case included a 61-year-old man, who had significant pain and paralysis in his left shoulder after contracting COVID-19. The patient was taken to the hospital with hemoptysis after two days of increasing cough prior to his first visit to the orthopedic clinic [69]. The patient was subjected to a lung CT scan on arrival at the emergency department. This revealed a minor, acute pulmonary embolism, and a tiny infiltration with lymphadenopathy in the right upper lobe. At the time, laboratory values were mostly normal. The patient was tested for COVID-19 due to his symptoms and was confirmed to be positive. After receiving remdesivir, he was discharged three days after his arrival in stable condition, with his respiratory status gradually improving. Several weeks later, the patient went to the emergency room with a gradual development of acute shoulder discomfort and major restrictions in active range of motion. A physical examination revealed deep forward flexion weakness and significant weakness with external rotation [69]. The rotator cuff on the MRI was found to be fully healed. Mild edema and atrophy of the supraspinatus and deltoid were the only notable findings. At that moment, the focus shifted to a neurologic cause for his extreme weakness [69]. EMG was also requested. This revealed a left brachial plexopathy that mainly affects the upper trunk, and continuous denervation. The activation of the infraspinatus, deltoid, and, to a lesser extent, the biceps brachii were severely reduced, with accompanying fibrillations [69]. 

Physiotherapy was started along with a domestic workout program. Both at rest and during action, his discomfort was kept minimal. Unfortunately, eight months after the beginning of symptoms, he was having severe difficulty in performing activities above shoulder height. The patient had neuralgic amyotrophy as a result of SARS-CoV-2 infection. He was first diagnosed with migrating neuralgia, which led to an emergency room visit for the acute shoulder pain resistant to oral and intravenous pain medicines and was accompanied by weakness and loss of mobility. The patient gradually improved over the course of a year, albeit he still had chronic weakness in his upper body at his last check-up [69]. The use of EMG investigations can aid in the identification of the illness as well as the delineation of healing. MRI tests may reveal edema inside the muscular bellies of afflicted muscles, which can help rule out associated disease. Recovery might take a long time, so cautious waiting is usually the best option, along with physiotherapy to encourage mobility [69].

## 6. Congenital and Genetic Conditions Less Frequently (<5 PubMed Hits) Associated with COVID-19

### 6.1. COVID-19 and Prader–Willi Syndrome (PWS)

Prader–Willi syndrome (PWS) is a multisystem illness with an incidence of 1 per 10,000–30,000 persons. Unless eating is externally managed, it is characterized by severe hypotonia with poor sucking and feeding problems in early infancy, followed by excessive eating and progressive development of morbid obesity in later infancy or early childhood [70]. In most cases, hypogonadism manifests itself as genital hypoplasia, poor pubertal development, and infertility in both males and females. A common reason of low stature is a lack of growth hormone (GH). Sleep disruption and type II diabetes mellitus are more common, as are unusual facial characteristics, strabismus, and scoliosis [70]. In contrast to the vast literature on the role in the development of severe COVID-19, there is no published data on the progression of COVID-19 in PWS, the most common form of syndromic obesity, which affects about 1 in 21,000 infants [71,72]. Early results were unexpected, considering that persons with PWS frequently have comorbidities, which significantly impact COVID-19 outcomes [71]. Furthermore, patients with PWS have difficulties in communicating their concerns and have a lower temperature when an infection begins, leading to a delay in diagnosis [73]. Finally, as many persons with PWS live in a community structure and social distancing might be problematic in adults with IDDs, the risk of SARS-CoV-2 infection is higher among them. Between March 2020 and January 2021, 288 adults participated in a study in France, with 38 of them diagnosed with COVID-19. The average age of the adult cohort was 34 years, and 58% of the participants were female, 89% were Caucasian, and 53% of patients were reported to have 15q11-q13 deletions [74]. The majority of patients (82%) were obese, with 55% suffering from severe obesity [74]. COVID-19 symptoms were widespread, but only one adult experienced anosmia, and 37% were asymptomatic. At the time of infection, 60% of patients were taking vitamin D supplements. Three adults were admitted to hospital with proven pneumonia; however, all symptomatic patients showed rapid, full recovery [74]. A poll was also conducted for 239 minors (young population), 13 of which had COVID-19. The average age was 9.6 years, with 38% of the participants being female, 85% Caucasian, and 62% reported having 15q11-q13 deletions. Obesity affected three children (23%), and two had sleep apnea syndrome. At the time of infection, all youngsters were living with their families at home. The majority (85%) were taking vitamin D supplements, and only one was on psychotropic medication (neuroleptics). The most notable conclusion of this study is that, despite multiple comorbidities, including obesity and diabetes, there was no severe form of COVID-19 in adults. The youthful age of the patients (mean age of 34 years) may explain this observation, as age is the most well-known risk factor for severe COVID-19 [71]. Another explanation for the absence of severe COVID-19 in adults with PWS could be the beneficial role of oxytocin (OXT), which appears to have special functions in immunologic defense: it suppresses neutrophil infiltration and inflammatory cytokine release, activates T-lymphocytes, and antagonizes the negative effects of ACE2 and other key COVID-19 pathological events [75]. The OXT system is defective in most individuals with PWS, and high plasma levels of OXT in people with PWS (perhaps due to overcompensation of a brain impairment) may provide a protective role against COVID-19 [75]. Furthermore, most of the patients were given vitamin D, which has been shown to protect against COVID-19 [76], even if there is no consensus on how to reduce the risk of severe COVID-19 development in individuals without a deficiency [77]. Furthermore, antidepressants were taken by a third of the individuals with PWS in the cohort, which may have aided in the course of COVID-19 [78]. Finally, in three studies [79,80,81], ten adults with diabetes (83% of people with diabetes) took Metformin, which reduced the mortality of hospitalized patients with COVID-19 and type II diabetes. However, it is difficult to know whether Metformin contributed to less severe diabetes or has a specific effect on COVID-19 in these studies. Despite multiple risk factors for severe COVID-19, such as obesity and diabetes, persons with PWS showed only mild or moderate COVID-19 in this study. The key explanations are their youthful age (34 years) and possibly additional defensive mechanisms yet to be discovered. Therefore, PWS cannot be regarded as a risk factor for severe COVID-19.

### 6.2. COVID-19 and Lysosomal Storage Disorders

Lysosomal storage disorders (LSD) are a group of 70 metabolic illnesses that are hereditary. While they are uncommon individually, they occur in 1 per 5000 live births as a group [82]. Affected people have multi-systemic involvement, which varies in severity and rate of illness development depending on the condition. Some LSDs have well-established therapies, including stem cell transplantation, parenteral enzyme replacement therapy (ERT), oral substrate reduction therapy or chaperone therapy. Furthermore, all affected individuals require supporting therapy, such as physiotherapy, among other things. Both ERT and supportive therapy are outpatient therapies that can be given in hospitals, in outpatient clinics or at home [83]. A study was conducted in Israel to evaluate the impact of the COVID-19 pandemic limitations on LSD patients [83]. Secondary goals included determining the influence of mood and behavioral changes on treatment adherence and determining the link between mood and cognitive and behavioral changes. A total of 48 patients were treated at four different medical institutions in Israel. The patient group included 29 men (60%) and 19 females (40%) with ages ranging from one to 42 years, with 46 patients (95%) under the age of 18 and a median age of 6.5 years. The participants had the following disorders: 27 patients (56%) with mucopolysaccharidosis (MPS): one with MPS type I (Hurler syndrome), two with MPS type II (Hunter syndrome), eight with MPS type IIIA (Sanfilippo A syndrome), 14 with MPS type IVA (Morquio syndrome), two with MPS type VI (Maroteaux–Lamy syndrome); seven with Pompe disease (15%), six with Niemann Pick type C disease (12%); two with Gaucher type 1 disease (4%); two with neuronal ceroid lipofuscinosis (NCL) (4%; where one had NCL5 and another had NCL6); one with Niemann Pick type A disease (2%); one with cystinosis (2%); one with I cell disease (mucolipidosis II) (2%); and one with Lysosomal acid lipase deficiency (2%) [83]. The SARS-CoV-2 infection was detected by PCR in four individuals, all of whom had been diagnosed with Sanfilippo A, and of which two were siblings. In terms of underlying treatment, 26 patients (55%) received intravenous ERT, 15 patients (31%) were on various oral medications such as chaperone therapy or supportive care, and seven patients (14%) received no treatment at all [83]. Before the COVID-19 outbreak, 24 patients (92%) were receiving intravenous infusions at the hospital regularly, while two patients (8%) were on home-therapy. Two more patients were sent home due to the epidemic. Thirty-one of the 38 patients (82%) who received regular therapy did not miss any of their appointments. Five patients (20%) experienced treatment disruptions while receiving ERT in the hospital setting: three patients missed an intravenous infusion due to their parents’ fear of going to the hospital, one patient missed an intravenous infusion due to logistic issues in his medical center and one patient missed an intravenous infusion due to low adherence, despite the COVID-19 pandemic. Seven of the 48 patients (14.5%) reported mood abnormalities and cognitive and physical impairment during the home quarantine. This cohort included 14 Morquio patients (29%), of which two patients missed one ERT infusion (14%), and one patient was not on ERT for reasons unrelated to COVID-19. Due to the difficulty in obtaining these services, nine Morquio patients (64%) reported missing physiotherapy treatments throughout the quarantine period. However, as these patients’ compliance with extra therapies was already low before the onset of COVID-19, this had no significant impact on them. As the subset of patients who missed ERT was small (n = 7), these findings should be interpreted cautiously and confirmed in larger cohorts [83]. Patients with worsened mood were 76 times more aggressive, 37 times more cognitively impaired, and 12.8 times more motorically impaired (all with *p*-value ˂ 0.001) [83]. Despite severe psychological stress as shown by aggression, cognitive function decrease, and motoric function degradation that were connected to general worsened mood, this research on LSD patients demonstrated rather undisturbed treatment throughout the COVID-19 epidemic in Israel [83]. Compared to prior investigations on LSD patients, such as those conducted on Sanfilippo individuals [84,85], it is difficult to determine whether aggressiveness or cognitive deterioration were accelerated by the psychological stress associated with the lockdown or were simply part of the disease’s natural history. The standard therapy of individuals with uncommon inborn metabolic defects with multisystemic involvement is complicated and burdensome for patients, especially during a worldwide pandemic [83].

### 6.3. COVID-19 and Hereditary Spherocytosis (HS)

Hereditary spherocytosis (HS) is a hemolytic condition that manifests itself in various ways, ranging from asymptomatic to chronic hemolysis. Due to genetic abnormalities in plasma membrane proteins, the membrane-cytoskeleton interface of red blood cells becomes unstable, increasing the likelihood of hemolysis induced by stress such as fever, hypoxia, or viral infections [86]. Due to the splenic evacuation of damaged red blood cells, which causes anemia, patients are treated with supportive transfusions or, splenectomy in the most severe cases [87]. Severance et al. described a case of a four-year-old boy who arrived in the hospital with two days of cough, congestion, and subjective fever and had a history of mild HS and sickle cell trait without prior splenectomy [88]. His oral intake was reduced with the presence of yellow discoloration in his eyes. The patient’s mother experienced similar symptoms. There were no known SARS-CoV-2 exposures. Upon admission at the emergency room, the patient was febrile and tachycardic, but otherwise hemodynamically stable. He had a palpable spleen tip slightly below the rib edge and scleral icterus, but no additional abnormalities were discovered. Furthermore, the chest X-ray was normal [88]. An acute hemolytic process was the cause of the anemia. His metabolic profile was adequate. A COVID-19 nasopharyngeal PCR test was conducted due to the fever, cough, and congestion. He was hospitalized and given a packed red blood cell infusion of 10 mL/kg (pRBCs). Fever prevented the completion of the transfusion, and he only got 6 mL/kg of cells as a result [88]. The fever that developed during the transfusion was thought to be caused by COVID-19 rather than a transfusion response. Due to his continually low hemoglobin, he was given a second transfusion, which he handled well. His hemoglobin levels improved, and his hemolysis indicators were on the mend. Hemoglobin levels were unchanged six hours later, indicating no additional hemolysis. He was sent home with orders to quarantine for 14 days. With cellular stress and splenic clearance, hereditary spherocytosis increases the risk of hemolysis. While hemolysis is a well-known consequence in individuals with HS and viral infections, it has only been documented in the setting of HCQ toxicity in COVID-19 patients [89]. In the case of COVID-19, this patient underlines the need to monitor individuals at risk of hemolysis regularly. There have been reports of SARS-CoV-2 infection in sickle cell disease patients, but none have addressed the danger in individuals with hemoglobin membrinopathies [88]. The need to monitor hemoglobin and hemolytic indicators in individuals with red blood cell membrane abnormalities and SARS-CoV-2 infection is highlighted in this case. Children with positive COVID-19 test findings and underlying hemolytic diseases should be examined for hemolysis if extensive COVID-19 testing is implemented. The degree of hemolysis in HS varies, and the initial hemolytic episode might happen in the context of a SARS-CoV-2 infection [88].

### 6.4. COVID-19 in Patients with Spina Bifida (SB)

Spina bifida (SB) is a congenital birth condition that is caused by the failure of the embryonic neural tube to close properly [90]. In the context of COVID-19, having a child with special needs has been identified as one factor causing greater parenting-related tiredness [91]. Youth with SB are a group that is particularly vulnerable to the consequences of the COVID-19 pandemic. Even under normal circumstances, adolescents, and young adults with SB (AYA-SB; 15–25 years of age) are in a period of transition that can be chaotic. They must navigate a complicated medical and self-care regimen, as well as several serious comorbidities. AYA-SB must also deal with new challenges to adherence and self-management due to the COVID-19 pandemic, such as disturbed social interactions, more time at home, and more time accessing technology and social media [92,93]. Parents of 18-year-olds with SB face various obstacles, including maintaining the medicine regimen, being concerned about the health, and being unsure about the independence of their children [94]. The influence of the COVID-19 pandemic on a national level of AYA-SB (15–25 years) and parents of SB youth under the age of 18 was the goal of one study. The analyses were exploratory, but the goal was to uncover requirements unique to SB adolescents and their families in vulnerable and frequently underserved communities. Exposure, Impact and Distress due to the pandemic were studied in detail [95]. AYA-SB (n = 298) and parents of children with SB (n = 200) were recruited to participate in this study by completing an anonymous online survey in English or Spanish. Participants supplied information on their demographic and medical conditions, and their access to and use of technology for mental health care. They also completed the COVID-19 Exposure and Family Impact Survey (CEFIS), which includes Exposure, Impact, and Distress subscales. Exploratory correlations and *t*-tests were used to look into possible links between CEFIS scores and demographic, medical, and access characteristics [96]. The results on the Exposure, Impact, and Distress subscales varied greatly. Demographic connections with Exposure differed for those with higher Impact and Distress (for example, White, non-Hispanic/Latino AYA reported higher rates of exposure [*p* 0.001]; AYA who identified with a minoritized racial/ethnic identity had a higher impact [*p* 0.03]). The most often appearing qualitative themes were effects on mental and behavioral health (n = 44), interference with medical care (n = 28), and interpersonal issues (n = 27) [96]. The findings of this study describe the influence of the pandemic on the lives of AYA-SB and parents of youth with SB, as well as the various ways in which this impact may affect different groups (e.g., those with more shunt revisions). Families with children who have SB should practice self-care, focus on SB self-management, and use digital tools for care and social connection [96].

### 6.5. COVID-19 and Hypothyroidism

Hypothyroidism is a disorder in which the thyroid produces and releases insufficient thyroid hormone into the bloodstream, resulting in slowed metabolism. Hypothyroidism, also known as an underactive thyroid, can induce fatigue, weight gain, and an inability to tolerate cold. Hormone replacement therapy has been the mainstay of treatment, so far. Since the start of the COVID-19 pandemic, a growing body of evidence suggests that SARS-CoV-2 infection affects several organs, with both short- and long-term consequences [97]. As thyroid hormones influence the formation and function of nearly every human cell, viral impacts on thyroid function can result in multisystem involvement (https://www.hindawi.com/journals/bmri/2016/9583495 accessed on 6 December 2021). SARS-CoV-2 may be directly involved in viral thyroiditis, according to a recent observation [98]. Low fT3 readings were linked to a higher rate of clinical decline, suggesting that SARS-CoV-2 directly influences thyroid function [98]. Mild symptoms were discovered in 84.3% of 191 COVID-19 patients (mean age 53.5 ± 17.2 years; 51.8% male), 12.6% had moderate symptoms, and 3.1% had severe symptoms [98]. Thyroid function was abnormal in 13.1% of the population [98]. In ten patients, TSH levels were abnormal, indicating subclinical thyrotoxicosis related to thyroiditis. Autoimmune thyroiditis was a likely factor in some of the patients’ subclinical hypothyroidism [98]. Ten of the patients exhibited low fT3, most likely due to a non-thyroidal disease condition. Low TSH (*p* = 0.030) and low fT3 (*p* = 0.007) were independently linked with lower SARS-CoV-2 PCR cycle threshold values and higher C-reactive protein, respectively. With increasing COVID-19 severity, there was a declining trend in fT3 (*p* = 0.032). COVID-19-related outcomes were less favorable in patients with low fT3 [98]. According to the study, thyroid dysfunction was seen in 13.2% of individuals with mild to moderate COVID-19. TSH levels were associated with reduced Ct values in RT-PCR tests. Systemic inflammation has been linked to low fT3 and fT3/fT4 ratios. Patients with fever frequently reported low TSH and fT3 values. Finally, a low fT3 level, associated with poor COVID-19 outcomes, may have a predictive value [98].

### 6.6. COVID-19 and Fragile X-Syndrome (FXS)

Fragile X-Syndrome (FXS) is a trinucleotide repeat abnormality that is the most common hereditary form of intellectual disability, with a global prevalence of 1 per 4000 in males and 1 per 5000–8000 in females. This gender-related illness can manifest as behavioral issues and delayed language development, akin to ASD [99,100]. Although it is not widely recognized as an immunological condition, there is evidence that patients with elevated trinucleotide repeats, such as those observed in FXS, have immune dysregulation and reduced cytokine responses [101,102]. Only one case of COVID-19 in a 46-year-old female patient with FXS has been documented since the outbreak [103]. In the study, the effect of her genetic condition on the clinical manifestation of COVID-19 was investigated. She had a history of a deep venous thrombosis (DVT) in her left lower leg, and other comorbidities such as hypertension, morbid obesity, type II diabetes, and asthma. FXS not only affects the central nervous system, but also causes other physiologic dysfunctions, such as alterations in immune-related indicators, according to a recent study. Patients with FXS have lower serum levels of numerous chemokines, including chemokine (C-X-C motif) ligand 10 (CXCL-10), a pro-inflammatory cytokine [104]. As a result, someone with FXS may have weakened immunity and be more vulnerable to infections. CXCL-10 has also been associated with a cytokine storm linked to more severe disease in COVID-19 patients [102,105,106]. Cytokine Storm Syndrome (also known as Cytokine Produce Syndrome), is a condition in which lymphocytes and macrophages release many pro- and anti-inflammatory mediators, resulting in uncontrolled local and systemic inflammation. Current research has revealed that the release of pro-inflammatory cytokines by macrophages and monocytes causes T-lymphocyte activation and, eventually, a cascade of enormous cytokine and chemokine production in SARS-CoV-2 patients [106,107]. Interleukin (IL)-1, IL-6, IL-10, tumor necrosis factor-alpha (TNF-a), interferon-gamma (IFN-γ), chemokine (C-C motif) ligand 2 (CCL-2), CXCL-9, and IL-8 are some of the additional cytokines found to be important in the pathophysiology of COVID-19 [102,106,108]. It has been hypothesized that a decreased CXCL-10 profile in individuals with FXS would indicate a protective effect against cytokine release and Cytokine Storm Syndrome in COVID-19 patients [103].

### 6.7. COVID-19 and Duchenne/Becker Muscular Dystrophy

One of the most frequent neuromuscular illnesses in children is Duchenne muscular dystrophy (DMD). Approximately 1 in 5000 newborn males worldwide are affected by this hereditary degenerative condition. Becker muscular dystrophy (BMD) is a milder type of dystrophinopathy than DMD [109]. DMD involves progressive weakening, loss of motor skills and eventually pulmonary and cardiac failure, with significant respiratory muscle weakness causing restrictive respiratory illness and a weak cough [110,111,112]. Corticosteroids have been established as the gold standard of therapy for DMD patients, so far [113,114,115]. DMD patients are a high-risk group in the recent global COVID-19 pandemic (https://www.cdc.gov/coronavirus/2019-ncov/need-extra-precautions/people-with-medical-conditions.html accessed on 6 December 2021). During the global outbreak, one Israeli study intended to evaluate the clinical presentations and outcomes of individuals with severe dystrophinopathies, infected with SARS-CoV-2 [116]. From March to December 2020, the cohort included DMD/BMD patients, who were tested and found to be infected with SARS-CoV-2 at Schneider Children’s Medical Center (SCMC) in Israel [116]. All patients were subjected to standard follow-up visits or phone/virtual contacts over the trial period. They received a survey about their SARS-CoV-2 infection status during routine visits or if they were hospitalized. Following their recovery, infected patients returned to the neuromuscular clinic regularly. Physical examinations, lung function tests, and chest X-rays were performed during these visits and compared to those acquired before becoming COVID-19 positive [116]. During the study period, seven (6%) of the 116 DMD/BMD patients followed at SCMC tested positive for COVID-19 [116]. The median age of SARS-CoV-2-infected DMD/BMD patients was 14 years (range 8–17), compared to 11 years (range 4–27) for all DMD/BMD patients monitored at SCMC, who were not infected (*p* = 0.26). Five of the COVID-19 positive patients were ambulatory, while the other two needed to be hospitalized [116]. One patient was admitted due to dyspnea and chest pain, while the others had headache and fever. The chest X-ray of the first patient revealed low lung volumes and small patch infiltrates. Due to the respiratory exacerbations in previous years, this patient was suspected of having pseudomonal co-infection and was treated with ceftazidime and azithromycin. He was also given dexamethasone (6 mg twice a day) for two days before returning to his regular prednisone medication [116]. Before admission, this patient required daily non-invasive ventilation. The length of time he used ventilation was increased each day while in hospital until one day before discharge. The other admitted patient’s chest x-ray indicated low lung volumes without infiltrates, and he did not require antibiotics or a dose adjustment of corticosteroids [116]. Both patients were admitted to dedicated negative-pressure air-conditioning rooms to reduce transmission to the medical team, as one was ventilated using non-invasive ventilation (NIV) and the other used cough-assist equipment. Both patients were released from the ventilation unit five days later [116]. Five of the seven COVID-19 DMD patients were obese, with the two symptomatic patients showing extreme obesity, with a BMI of 33 kg/m^2^ (>99% for age). All DMD/BMD COVID-19 patients had a mean and median BMI of 26.7 and 31.2 kg/m^2^, respectively [116]. Finally, all symptomatic individuals recovered without any long-term consequences. There was no evidence of a severe course of disease in adult individuals with chronic lung disease and obesity. The hypothesis was that their muscular illness might have caused more social distancing due to fear of the disease [116].

### 6.8. COVID-19 and Neuromyelitis Optica Spectrum Disorder

Neuromyelitis optica spectrum disorder (NMOSD) is a rare central nervous system antibody-mediated illness [117]. It was unclear, until as recently as 2004, whether NMOSD was a distinct illness or simply a more severe variant of ‘optico-spinal’ multiple sclerosis (MS), when the potential antigenic target, the aquaporin-4 water channel, was discovered, and the two disorders could be consistently differentiated using aquaporin-4 antibodies (AQP4-Abs) [118,119]. NMOSD clustering in families is uncommon but recognized, indicating a complicated genetic predisposition. A recent whole-genome sequencing initiative discovered genetic variations in the major histocompatibility region that may play a role in the genesis of NMOSD [120,121]. One in every four individuals with AQP4-Ab positive NMOSD has also another autoimmune illness, such as myasthenia gravis, systemic lupus erythematosus (SLE), Sjogren’s syndrome, or celiac disease [122,123,124]. Patients with NMOSD are sensitive to developing COVID-19 owing to immunosuppressive medication [125]. In individuals with NMOSD, long-term immunosuppression is the basis for treatment, which might hypothetically raise the risk of infections such as SARS-CoV-2 [126]. Paybast et al. reported a case study of a young 25-year-old female with a history of NMOSD, who had two separate episodes of COVID-19 while receiving rituximab therapy [125]. Due to the close contact with a COVID-19 patient, she was recommended for a COVID-19 screening test in April 2020. The COVID-19 test showed no upper respiratory tract discomfort symptoms. The neurological examination was significant for a score of two on the Expanded Disability Status Scale. Due to COVID-19 fear and the proximity of the next rituximab infusion, the patient had serologic testing, which confirmed positive SARS-CoV-2 IgM and IgG. Her rituximab treatment was postponed until September 2020. The patient returned in November 2020 with a five-day history of severe chills, fever, and shortness of breath. Her chest computed tomography (CT) revealed bilateral ground-glass infiltrates. The patient was hospitalized and given conventional COVID-19 medication and non-invasive oxygen supplementation [125]. The patient steadily recovered and was discharged two weeks later. The patient’s symptoms, however, did not totally disappear. Unfortunately, the patient’s respiratory problems worsened three weeks later, necessitating readmission. Due to respiratory distress, the patient was brought to the critical care unit and required breathing assistance. Due to the severity of COVID-19-related respiratory involvement, the therapy began with therapeutic plasma exchange (TPE). The patient’s symptoms gradually improved, and she was transferred to the ward five days later. Her hemodynamics were stable, and her chest CT indicated that the lung infiltration had cleared significantly. On the 31st day, she was allowed to return home [125]. As there was a long interval between the two episodes, the reported case described both persistent viral shedding (the second scenario of readmission) and a true reactivation of the infection (the first scenario of admission). Unlike earlier accounts, this patient was asymptomatic throughout the first episode, and the second episode was worsened by respiratory distress due to continuous viral shedding [125]. In contrast, there is no evidence that patients taking immunosuppressive drugs are at a higher risk of COVID-19 complications [127]. Although rituximab appears to decrease the likelihood of severe SARS-CoV-2 infection, a muted vaccination response, and a viral reactivation, observational studies on MS and COVID-19 have not found that rituximab plays a role in COVID-19 occurrence [128,129]. Taking these factors into account, this case emphasizes the significance of paying extra attention to vulnerable groups such as patients with NMOSD during the COVID-19 pandemic, especially in the event of COVID-19 recurrence [125]. 

## 7. Multi-Omics-Based Predicted Congenital Anomalies and Genetic Diseases and Their Associations with COVID-19

Using a multi-omics approach, Barh and colleagues predicted for the first time the association between congenital anomalies and genetic disorders and COVID-19 [5]. They predicted various COVID-19 associated symptoms, conditions, and possible long-term complications with up to 92% accuracy. According to their analysis, 57 various congenital and genetic diseases could be associated with COVID-19. Based on the cut off values, the predicted 24 important conditions associated with COVID-19 are congenital hemolytic anemia, dyserythropoietic anemia, thalassemia, congenital disorders of glycosylation or congenital porphyria, occipital horn syndrome, gangliosidosis GM1 type 3, Sandhoff disease, beta-galactosidase-1 deficiency, Aicardi-Goutieres syndrome, DS, Atkin syndrome, cleft palate or bilateral cleft lip, congenital coagulation defects, genital organ defects, ceroid storage disease, aspartylglucosaminuria, adrenoleukodystrophy, familial Paget’s disease of bone, Asperger syndrome, ASD, head morphology, Sotos syndrome, arthrogryposis, limb defects, CF, rhizomelic chondrodysplasia punctate, fetal hemoglobin quantitative trait locus, hereditary elliptocytosis, and familial erythrocytosis. As per their prediction, the 33 low confidence conditions are bare lymphocyte syndrome, hemophagocytic lymphohistiocytosis, pontocerebellar hypoplasia type 1, Canavan disease, Aicardi syndrome, Schwartz Jampel syndrome type 1, Melnick-Needles syndrome, hereditary connective tissue disorder, lower limb spasticity/spastic paraplegia, sclerosteosis, Clayton-Smith Donnai syndrome, alpha-1 anti-trypsin deficiency, type 1 plasminogen deficiency, Polydactyly Zechi Ceide syndrome, congenital hypoplasia of femur, adducted thumb, sickle cell anemia, congenital anomalies of the eye, congenital euryblepharon, Kabuki syndrome eyelids, lissencephaly, Leukomalacia, xeroderma pigmentosum, Rothmund Thomson syndrome, parental lifespan, CHD, adrenal hypoplasia congenital, tetraploidy, shallow anterior chamber of the eye, Treacher Collins syndrome, disorders of tooth development, and hereditary spherocytosis. However, as the prediction accuracy percentage was based on the phenome data and the congenital/genetic disease associated with COVID-19 data is not available in any structured form, Barh et al. predicted this association with an accuracy of 40% [5]. However, increasing COVID-19 phenome data indicate that their prediction accuracy on COVID-19 and congenital/genetic disorder association would be higher than calculated and more focus should be placed on understanding the effect of SARS-CoV-2 infections in persons with existing congenital/genetic disorders or if congenital/genetic conditions arise as a long-term consequence of COVID-19. 

If we consider that congenital/genetic disorders could have long-term consequences of COVID-19, they may or may not be expressed in the immediate offspring and may take generations to manifest themselves. In principle, to develop a congenital/genetic disease, the genetic material (human genes, chromosomes) must be disrupted to express the disease phenotype. If the predictions of Barh et al. [5] are correct and if we consider their “genetic remittance” hypothesis, where the SARS-CoV-2 genetic material may integrate in and/or disrupt the human genome, some human genes or chromosomes may be malfunctional, which would result in congenital/genetic disorder development in the offspring. Although, there are Genome-Wide Association Studies (GWAS) to understand the COVID-19 susceptible or resistance loci in humans [130,131,132], so far there is not much information available on whether the SARS-CoV-2 genetic material is integrated in the genome or disrupting it. However, an in vitro analysis suggested that transcribed SARS-CoV-2 RNA may potentially integrate into the genome of cultured human cells [133] indicating the possibility of Barh et al.’s hypothesis. However, some controversy has been associated with the potential genome integration of SARS-CoV-2, which requires more research on the integration process and its affects and consequences (https://www.science.org/content/article/coronavirus-may-sometimes-slip-its-genetic-material-human-chromosomes-what-does-mean accessed on 28 December 2021).

Barh et al. predicted that reproductive system diseases could be long-term consequences of COVID-19 [5]. Reports suggest that SARS-CoV-2 is detected in testicular samples and is associated with impaired testicular function and spermatogenesis [134]. Similarly, a declined ovarian reserve and reproductive endocrine disorders are found in SARS-CoV-2 infected women [135]. Therefore, COVID-19 seems to affect the reproductive health of both males and females, also influencing fertility [136], which may also lead to birth defects and congenital abnormalities in the future. Equally, COVID-19 associated congenital disorders may arise through alternative mechanisms, like those discovered for Zika virus (ZIKV). Therefore, it is also necessary to investigate whether transplacental transmission and placental insufficiency occurring for ZIKV [137,138] can also be detected for SARS-CoV-2.

A summary of our discussed congenital anomalies and genetic diseases and their associations with COVID-19 is presented in Table 1.

## 8. COVID-19 during Early Pregnancy

It is known that miscarriage, congenital anomalies, and fetal growth restriction represent some of the severe complications of the spectrum of infections by SARS-CoV, MERS-CoV, and SARS-CoV-2 during pregnancy [176]. It was pointed out that pregnant women tested positive for COVID-19 are more likely to be hospitalized [177]. Moreover, SARS-CoV-2 infections of women at late pregnancy is linked to increased rates of adverse birth outcomes, with preterm birth being the most common adverse pregnancy outcome (in addition to preeclampsia, cesarean, and perinatal death) in hospitalized mothers with coronavirus infections, who also had pneumonia [2]. As far as the consequences of SARS-CoV-2 infection in early pregnancy are concerned, it was hypothesized that the infection itself and the utilization of antiviral drugs for COVID-19 containment might be associated with an increased risk for congenital neurodevelopmental anomalies in newborns [178]. The neural tube defects (NTDs) leading to severe malformations of the spinal cord (spina bifida) or brain (anencephaly, encephalocele, hydrocephalus) [178] are among the common severe congenital malformations developed in early pregnancy complicated by viral infections [179,180]. Furthermore, a higher incidence of mental illnesses, such as attention deficit disorders, ASD, and schizophrenia were reported for offspring of women, who were pregnant during influenza pandemics [181,182,183,184,185]. It has also been reported that infants delivered by mothers, who were infected by SARS-CoV-2 during pregnancy might show decreased motor function, communication, and social development [186]. 

The capability of SARS-CoV-2 to cross the placental barrier [186] and the blood–brain barrier [187] suggests that this virus can cause some adverse effects and can be associated with NTD pathogenesis [178]. Since the ACE2, the host cell receptor interacting with the SARS-CoV-2 spike protein (S) and S protein proteases (furin and the transmembrane serine protease TMPRSS2, which are needed for viral S protein priming) are expressed in early gametes, zygotes, and four-cell stage embryos [188] and since developing human embryos also possess the machinery necessary for viral internalization and replication, it is possible that the in utero maternal to fetal transmission of SARS-CoV-2 might occur during early pregnancy [188]. In a recent study of an early miscarriage in a SARS-CoV-2 maternal infection, prominent damage of the placenta and fetal organs, such as lung and kidney, was reported, indicating that congenital SARS-CoV-2 infection during the first trimester of pregnancy is possible and that coronaviruses target fetal organs [189]. Although it was suggested that congenital SARS-CoV-2 infection during early pregnancy could potentially trigger neurodevelopmental complications [178], no supporting evidence has been reported, so far. Furthermore, it was established that pregnant women are less likely to contract SARS-CoV-2 than the general public [189], and that the likelihood of SARS-CoV-2 vertical transmission is low [190,191,192,193,194,195]. For example, only 5.7% of 176 neonatal SARS-CoV-2 infections cases were classified as confirmed congenital infections [196]. In addition, although SARS-CoV-2 infection was shown to cause significant placental pathology, the actual consequences of COVID-19 for early pregnancy are not clear, and reported data indicate that in comparison to other respiratory viral outbreaks, the effects of SARS-CoV-2 in early pregnancy are less severe [197]. 

Published research indicated that viral infection during early pregnancy, along with various antiviral drugs, was linked to an elevated risk of neonatal neurological congenital abnormalities [178]. SARS-CoV-2 appears to cross both the placental barrier, based on detection of viral IgM in newborns hours after birth, and the blood–brain barrier (BBB) by the presence of the virus in the cerebrospinal fluids [185]. There are various issues regarding the use of antiviral medication to confine SARS-CoV-2 and minimize virus-related consequences, including pneumonia. 

Although no effective drugs to combat SARS-CoV-2 have been discovered, several antiviral and anti-inflammatory therapies developed for other viral infections and pathologies have been repurposed for COVID-19. For example, favipiravir, previously used against influenza virus, remdesivir, developed for Ebola virus disease; and dolutegravir/lamivudine/tenofovir against human immunodeficiency viruses (HIV), have been evaluated for COVID-19. However, as the implications of these drugs on pregnancy, mainly in the first trimester, have not been investigated, there is serious concern that they might cause adverse birth outcomes. Favipiravir has been linked to birth abnormalities and is not recommended for women who are or may be pregnant [198]. Despite that, with the assistance of the Japanese government, this medication was extensively used to treat COVID-19 in approximately 40 nations by the end of 2020 and in numerous developing and source-constrained countries due to its wide availability. However, the drug has not been licensed or approved by either the Japanese government or the FDA for its use to combat COVID-19 [2]. Dolutegravir, an efficient HIV drug, has also been evaluated for the treatment of COVID-19 patients in low- and middle-income economies. However, it was shown that dolutegravir increased the frequency of neural tube defects, and their prevalence three-fold [199]. Additionally, dolutegravir administration during pregnancy resulted in a significant increase in external physical abnormalities in newborns (9 cases in 1000 births) [199].

**Figure 3:**
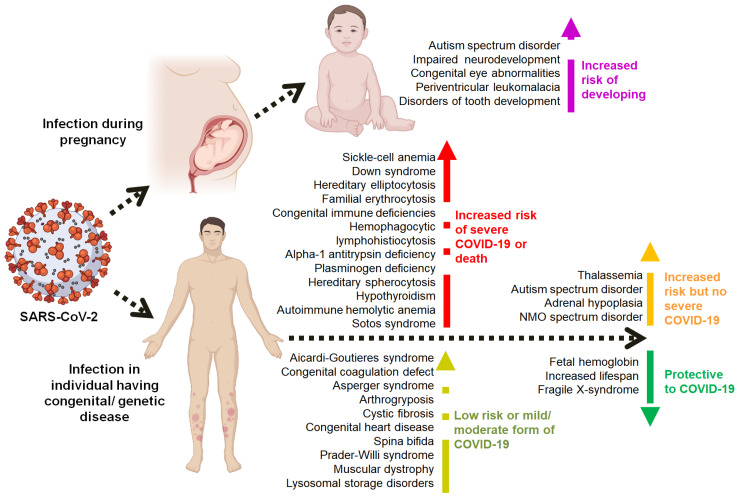
Summary of associations, disease–disease interactions, and impacts of COVID-19 in congenital anomalies and genetic disorders.

A graphical illustration of COVID-19 impacts on congenital anomalies and genetic diseases discussed in this review is presented in Figure 3.

## 9. Conclusions

To the best of our knowledge, this is the first comprehensive review describing the association between COVID-19 and various congenital anomalies and genetic diseases. The data assembled here provide strong support for the idea that COVID-19 may induce long-term congenital anomalies among newborns, either through infections or through therapeutic intervention. Such facts become much more significant in impoverished and resource-constrained societies whereby prenatal screening, particularly during the early stages of pregnancy, is essentially non-existent. Assessing the mechanistic connections between the host genetic profile with COVID-19 is thus critical to ascertain biomarkers for those at increased risk, which may also reveal potential candidates for intervention. Furthermore, as observed from various studies, some congenital disorders present high-risk for developing severe COVID-19. CHD, CF, and DS would be those. These disorders already include some comorbidities related to the structure and function of respiratory and cardiovascular systems, leading to severe pneumonia. Other congenital disorders like SB and PWS cannot be considered risk factors for severe COVID-19 but rather cause psychological burdens to patients. Low fT3 concentration found in patients with hypothyroidism may have a predictive value for this type of disease, but without the occurrence of any severe cases of COVID-19. 

On the other hand, due to the lack of data on FXS patients with COVID-19 and lack of understanding of the cytokines involved in the pathophysiology of SARS-CoV-2 infection, it is difficult to draw any conclusions about the relationship between FXS and its protective effect against severe clinical outcomes in COVID-19 patients. According to the literature, individuals suffering from DMD, and BMD recovered without any long-term consequences, excluding muscular dystrophy as a high-risk for developing a severe form of COVID-19. The severity of SARS-CoV-2 infections in patients with thalassemia, LSD and ASD is low, however, there was an impact on mental health. On the other hand, the incidence in patients with CF, AIHA, and HS can be considered moderate to high due to hemolysis in the case of HS and AIHA or due to transplants in the case of CF. Immunosuppressive medication in patients with NMOSD might hypothetically increase the risk of infections such as SARS-CoV-2. However, it was shown that this kind of therapy was not found to play a role in COVID-19 occurrence. Concerning SARS-CoV-2 infections during early pregnancy, it was suggested that congenital infections during this period could potentially trigger neurodevelopmental complications; however, no supporting evidence has been reported so far. It was also established that pregnant women are less likely to contract COVID-19 compared to others, and that the likelihood of SARS-CoV-2 vertical transmission is low.

## Figures and Tables

**Table 1:** A snapshot of reported associations of COVID-19 with congenital anomalies and genetic diseases.

Disease	Incidence	Association	References (DOI)
Sickle-cell anemia	Moderate	Increased risk of developing severe COVID-19 symptoms including acute chest syndrome, vasoocclusive crises, and death.	[139,140,141,142]
Thalassemia	Low	In risk population but does not increase the severity of COVID-19. However, pulmonary microembolism is reported and death occurred due to another comorbidity.	[48,143,144,145]
Aicardi-Goutieres Syndrome (AGS)	Low	Mostly asymptomatic or mild or shows rash on cheeks and arms. Post-COVID-19 generalized panniculitis is observed and the *SAMDH1* gene could be a potential link between COVID-19 and AGS	[146,147]
Down Syndrome (DS)	Moderate	DS exhibits a higher risk of COVID-19 severity and 10 times higher mortality of SARS-CoV-2 infections.	[33,148]
Congenital coagulation defect	Low	May not be a risk factor for increased severity from COVID-19. Hypercoagulability may have a protective role against SARS-CoV-2 infection.	[149,150]
Genital organ defects	Moderate	Although the genetic or congenital condition of genital organ defects is not reported, testicular spermatogenesis dysfunction and reduced sperm count are frequent after SARS-CoV-2 infection.	https://www.europeanreview.org/article/24682 (accessed on 14 February 2022).
Asperger’s syndrome (AS)	Low	AS shows mental health and behavioral issues in COVID-19 patients.	[151]
Autism Spectrum Disorder (ASD)	Low	SARS-CoV-2 may impair brain development via cytokine storm during pregnancy increasing the risk of ASD. ASD increased vulnerability to COVID-19 in children and affected their behavior.	[22,152,153]
Sotos syndrome	Low	Pericardial effusion after infection with SARS-CoV-2 is reported.	[154]
Arthrogryposis	Low	Shows mild or no symptoms of COVID-19.	[155]
Cystic fibrosis (CF)	Low	CF is not a high-risk, however, CF patients with low lung function or transplants may show severe symptoms of COVID-19.	[16,156]
Fetal hemoglobin quantitative trait locus	High	Increased level of fetal hemoglobin may prevent hypoxia and cure respiratory distress syndrome in COVID-19.	[157]
Hereditary elliptocytosis	Low	May show varying severity and risk of hemolysis in COVID-19.	[88]
Familial erythrocytosis	Low	The presence of erythrocytosis increases the risk of thrombosis in COVID-19.	[158]
Bare lymphocyte syndrome (BLS)/Congenital immune deficiencies (CID)	Low	Convalescent plasma therapy may be effective in CID patients suffering from COVID-19.	[159]
Hemophagocytic lymphohistiocytosis (HL)	Low	HL may be a secondary event or a risk factor of severe COVID-19	[160,161]
Aicardi syndrome/Malformations in brain	Low	Differences in neurodevelopment are observed in infants of six months infected by SARS-CoV-2 at the fetal stage. Long-term observation is required.	[162,163,164]
Alpha-1 antitrypsin deficiency (AAD)	High	AAD patients show worse outcome due to TMPRSS2 being activated more easily. They also have an increased risk of coagulation disorder and severe acute lung injury from COVID-19.	[165]
Plasminogen deficiency	Moderate	Low plasminogen level exhibits 12-fold higher mortality from COVID-19.	[166]
Congenital anomalies of the eye/congenital euryblepharon	Low	Eye abnormalities are observed in newborns infected by SARS-CoV-2 at the fetal stage.	[167]
Leukomalacia	Low	Periventricular leukomalacia is reported in newborns infected by SARS-CoV-2 at the fetal stage.	[164]
Adrenal hypoplasia (AH)	Low	AH patients undergoing glucocorticoid replacement therapy for adrenal insufficiency are vulnerable to developing severe complications from COVID-19.	[168]
Disorders of tooth development	Low	A fetus may be at high risk for enamel defects due to the stress of COVID-19 during pregnancy. Tooth loss is observed in severe COVID-19.	[169,170]
Hereditary spherocytosis (HS)	Moderate	HS patients show an increased risk of hemolysis and splenomegaly due to COVID-19.	[88,171]
Lifespan	Not Known Available	Genetic polymorphisms that are linked to longer lifespans are significantly associated with a low risk of SARS-CoV-2 infection and hospitalization.	[172]
Congenital heart disease (CHD)	Low	CHD are of low or moderate risk, which may develop hemodynamic abnormalities upon SARS-CoV-2 infection but has no impact on mortality.	[173,174,175]
Spina Bifida (SB)	Low	Impact on mental health	[90,91,92,93,94,95,96]
Fragile X-Syndrome (FXS)	Low	Decreased CXCL-10 as protection against Cytokine Storm Syndrome	[99,100,101,102,103,104,105,106,107,108]
Prader-Willi Syndrome (PWS)	Low	Youthful age of the cohort had a positive impact, so far	[70,71,72,73,74,75,76,77,78,79,80,81]
Hypothyroidism	Low to Moderate	Low fT3 level can lead to poor outcomes	[97,98]
Duchenne/Becker Muscular Dystrophy (DMD/BMD)	Low	All symptomatic individuals recovered without any long-term consequences	[109,110,111,112,113,114,115,116]
Lysosomal Storage Disorders (LSD)	Low	Impact on mental health	[82,83,84,85]
Autoimmune Hemolytic Anemia (AIHA)	Moderate	Hemolysis	[23,24,25]
NMO Spectrum Disorder	Low	Readmission	[117,118,119,120,121,122,123,124,125,126,127,128,129]

## Data Availability

Data are contained within the article.
